# Analysis of risk factors for perifocal oedema after endovascular embolization of unruptured intracranial arterial aneurysms

**DOI:** 10.1515/raon-2015-0044

**Published:** 2015-11-27

**Authors:** Snezana Lukic, Slobodan Jankovic, Katarina Surlan Popovic, Dragic Bankovic, Peter Popovic, Milan Mijailovic

**Affiliations:** 1 Department for Interventional Neuroradiology, Clinical Center, Kragujevac, University of Kragujevac, Serbia; 2 Department for Clinical Pharmacology, Clinical Center, Kragujevac, Serbia; 3 Clinical Radiology Institute, University Medical Centre Ljubljana, Ljubljana, Slovenia; 4 Faculty of Natural Sciences and Mathematics, University of Kragujevac, Serbia

**Keywords:** intracranial aneurysms, endovascular embolization, perianeurysmal oedema, hypertension

## Abstract

**Background:**

Endovascular embolization is a treatment of choice for the management of unruptured intracranial aneurysms, but sometimes is complicated with perianeurysmal oedema. The aim of our study was to establish incidence and outcomes of perianeurysmal oedema after endovascular coiling of unruptured intracranial aneurysms, and to reveal possible risk factors for development of this potentially serious complication.

**Methods:**

In total 119 adult patients with endovascular embolization of unruptured intracranial aneurysm (performed at Department for Interventional Neuroradiology, Clinical Center, Kragujevac, Serbia) were included in our study. The embolizations were made by electrolite-detachable platinum coils: pure platinum, hydrophilic and combination of platinum and hydrophilic coils. Primary outcome variable was perianeurysmal oedema visualized by magnetic resonance imaging (MRI) 7, 30 and 90 days after the embolization.

**Results:**

The perianurysmal oedema appeared in 47.6% of patients treated with hydrophilic coils, in 21.6% of patients treated with platinum coils, and in 53.8% of those treated with mixed type of the coils. The multivariate logistic regression showed that variables associated with occurrence of perianeurysmal oedema are volume of the aneurysm, hypertension, diabetes and smoking habit. Hypertension is the most important independent predictor of the perianeurysmal oedema, followed by smoking and diabetes.

**Conclusions:**

The results of our study suggest that older patients with larger unruptured intracranial aneurysms, who suffer from diabetes mellitus and hypertension, and have the smoking habit, are under much higher risk of having perianeurysmal oedema after endovascular coiling.

## Introduction

During the past decade method of endovascular embolization became a treatment of choice for the management of unruptured intracranial aneurysms, which are increasingly being diagnosed in the era of modern imaging methods. Endovascular embolization has been validated as a minimally invasive and effective treatment that can prevent rupture of an aneurysm and intracranial haemorrhage, with shortening of hospital stay and faster patient recovery compared to the surgical treatment. Further step in development of this therapeutic procedure was design of bioactive coils, which are very effective, but sometimes followed by perianeurysmal oedema. There are a few published case reports linking these coils with development of the perianeurysmal edema^1^–^7^, but we are still far away from complete understanding of this phenomenon. Almost identical post embolization reactions could be seen after use of bare platinum coils^8^,^9^, and it is not clear whether the per aneurysmal oedema is an adverse reaction to the coils itself, or just to specific type(s) of the coils.

The perianeurysmal oedema could clinically present itself with headache, lethargy, confusion, meningismus, seizures, disorders of visual function and/or cranial nerve(s) palsy.^1^,^2^,^4^,^5^,^7^ Majority of the reported perianeurysmal oedema cases appeared after coiling large aneurysms^6^, but oedema was also recorded after embolization of small aneurysms.^9^ From the cases described so far, we could conclude that the clinical appearance, the time frame of oedema occurrence and the final patient outcome are highly variable.

Perianeurysmal oedema is relatively rare complication that follows embolization of intracranial aneurysms. Most of the patients with this complication are clinically inconspicuous, asymptomatic or with nonspecific symptoms, which leads to underestimation of the incidence and importance of this complication.^5^ We are currently unaware of any risk factors that could predetermine appearance or severity of the perianeurysmal oedema after endovascular coiling.^10^–^13^

The aim of our study was to establish incidence and outcomes of perianeurysmal oedema after endovascular coiling of unruptured intracranial aneurysms, and to reveal possible risk factors for development of this potentially serious complication.

## Material and methods

### Study design

Our study was designed as case series, with an embedded case-control study. The case series consisted of patients who underwent endovascular embolization of unruptured intracranial aneurysm; those of them who developed perianeurysmal oedema were then considered to be the „cases“, and the rest of them were classified as „controls“. The cases and controls were not matched.

The study was approved by the Clinical Center Kragujevac Medical Ethics Committee (No. 01/8642) and was carried out according to the Declaration of Helsinki as well as we have followed the relevant guidelines in this investigation. All patients signed informed consent.

### Study population

In total 119 patients (of both sex and older than 18 years) with endovascular embolization of unruptured intracranial aneurysm were included in our study. The embolizations were performed by experienced interventional neuroradilogists (more than 200 performed embolizations each) at Department for Interventional Neuroradiology, Clinical Center, Kragujevac, Serbia, from January 2008 to December 2012. The embolizations were made by etachable platinum coils: pure platinum, hydrophilic and combination of platinum and hydrophilic coils. In order to prevent extrusion of the coils from aneurysms with broad neck, self-expanding stents were used.

### Study variables

**FIGURE 1.**
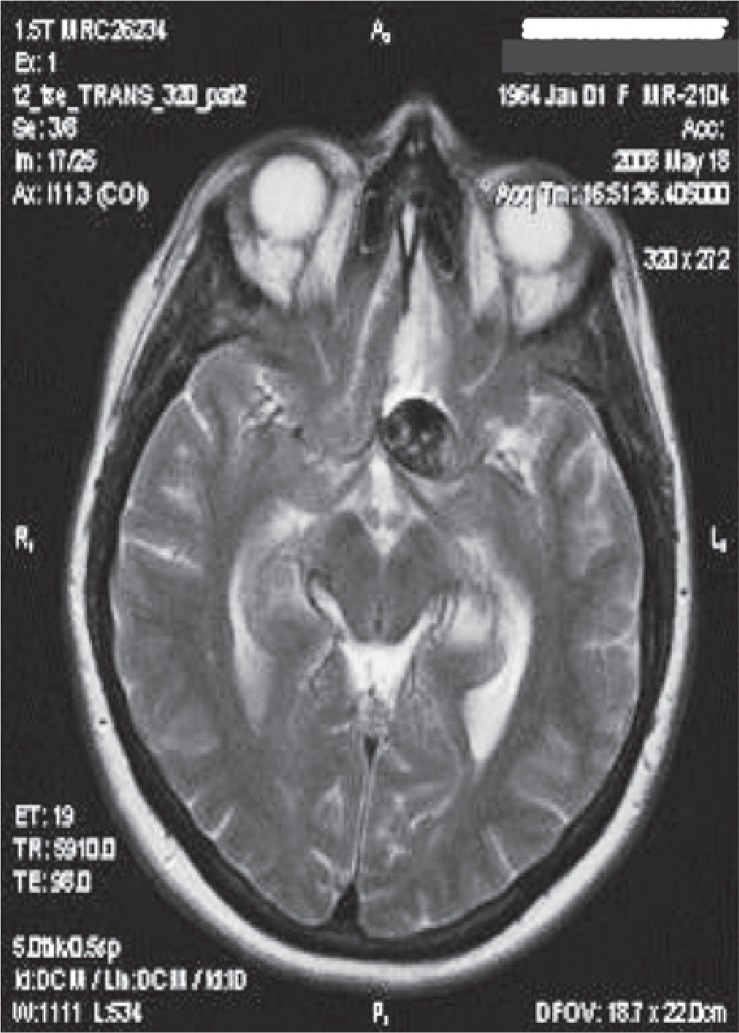
T2W axial MR tomogram of a patient showing perifocal oedema in frontal region after embolization of the left ophtalmic artery aneurysm.

**FIGURE 2.**
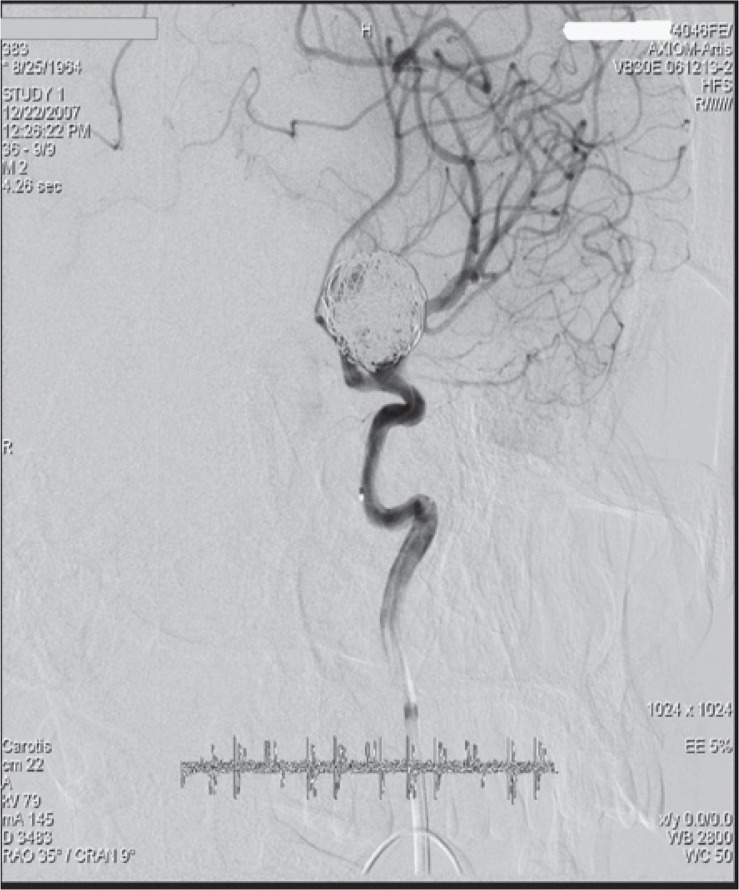
Digital subtraction angiography (DSA) of a patient after embolization of the unruptured left ophtalmic artery aneurysm, 13 × 10mm in diameter.

**FIGURE 3.**
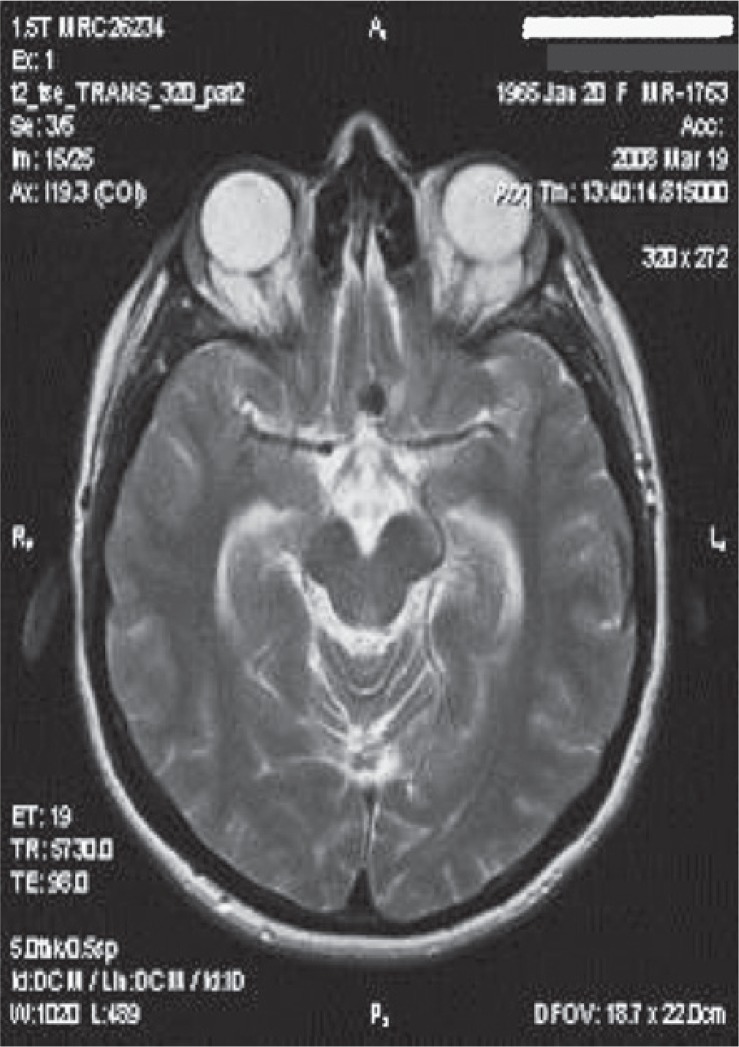
T2W axial MR tomogram of a patient showing minimal perifocal oedema in left frontal region after embolization of the anterior communicating artery aneurysm 5 × 5 mm in diameter.

**FIGURE 4.**
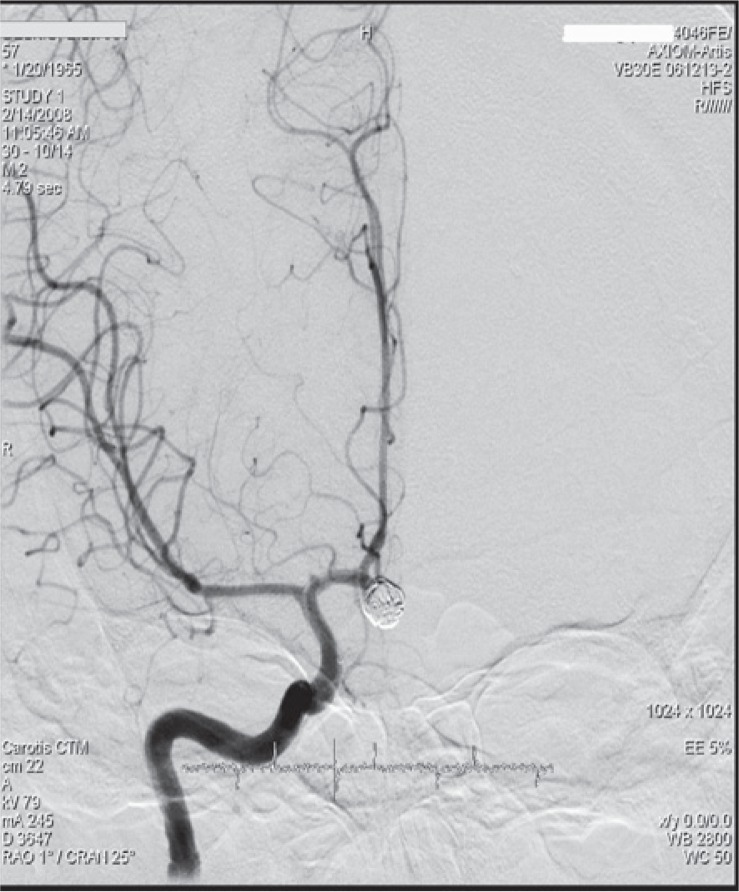
Digital subtraction angiography (DSA) of a patient after endovascular embolization of the unruptured anterior communicating artery aneurysm, 5 × 5 mm in diameter.

Primary outcome variable was perianeurysmal oedema visualized by magnetic resonance imaging (MRI) imaging 7, 30 and 90 days after the embolization, using standard T1 weighted (W), T2W and fluid attenuated inversion recovery (FLAIR) sequences (Figures 1–4). There are predictor variables that were followed in the study: age of the patients, sex, arterial blood pressure, serum cholesterol level, size and location of the aneurysm, type of the coils, patient medication and smoking habits.

### Statistical analysis

The study data were primarily described using medians, means and standard deviations for continuous variables, and percentages and odds for categorical variables. Normality of the data distribution was checked by Kolgomorov-Smirnof test. The differences in values of predictor variables among the study groups (patients with and without perianeurysmal oedema) were tested for significance by Student’s T-test (for continuous variables) or by Chi-square test (for categorical variables). Univariate and multivariate logistic regression analyses were used to study the relationship between occurrence of perianeurysmal oedema and the predictor variables. The differences among the study groups were considered to be significant if probability of null hypothesis was less than 0.05.

## Results

The perianeurysmal oedema was observed by NMR imaging in 45 of 119 patients (37.8%). However, only 8 patients (6.7%) developed symptomatic oedema, with transient headache and malaise. The symptoms withdrew spontaneously in the next 48 hours. The characteristics of the patients with and without the perianeurysmal oedema are shown in Table 1. Location of the aneurysms was similar in the both groups (p = 0.268), but type of the coils determined occurrence rate of the perianeurysmal oedema: it appeared in 47.6% of patients treated with hydrophilic coils, in 21.6% of patients treated with platinum coils, and in 53.8% of those treated with mixed type of the coils. However, the only significant difference in rate of perianurismal oedema was between the patients treated with platinum coils and the patients treated with mixed type of coils. All of the patients with symptomatic oedema were treated with mixed type coils.

**FIGURE 5.**
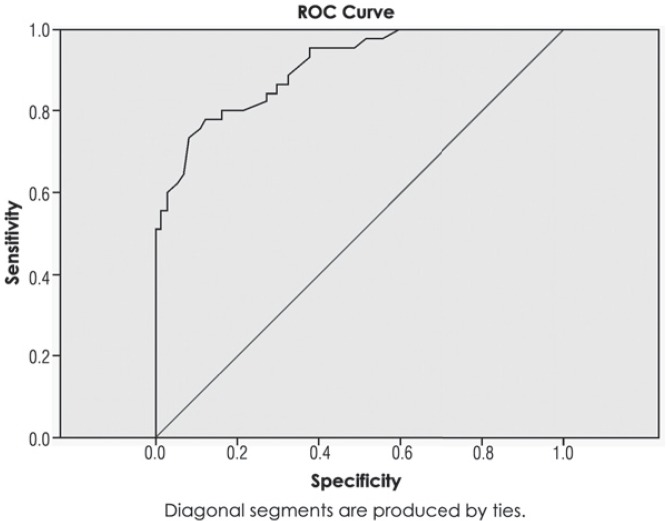
The receiver-operator curve (ROC) for volume of the intracranial aneurysms and occurrence of the perianeurysmal oedema after endovascular embolization.

From the Table 1 one could see that the higher the volume of an aneurysm, the perianeurysmal oedema is more frequent. If a Receiver-Operator Curve (ROC) is constructed for volume of the aneurysms and occurrence of the oedema after endovascular embolization (Figure 5), it turns out that growth of volume of aneurysm for 1 mm^3^ increases risk of perianeurysmal oedema for 1.3% (AUROC = 0.908, p = 0.0005). If a cut-off value of 174 mm^3^ is taken into account, the volume of an aneurysm could predict occurrence of perianeurysmal oedema with sensitivity of 80.08%, and specificity of 83.8% (positive predictive value is 75.0%, and negative predictive value is 87.3%).

The results of univariate and multivariate binary logistic regression for occurrence of perianurysmal oedema as dependent variable are shown in Table 2. After adjustment for other followed variables, the multivariate regression showed that variables associated with occurrence of perianeurysmal oedema are volume of the aneurysm, hypertension, diabetes and smoking habit. Hypertension is the most important independent predictor of the perianeurysmal oedema (30-fold increased risk), followed by smoking and diabetes (about 5-fold increased risk with each of the variables).

## Discussion

Most of previous reports on perianeurysmal oedema after endovascular coiling described patients with developed clinical picture.^4^,^5^,^9^ However, perianeurysmal oedema occurs also in asymptomatic and patients with mild non-specific symptoms, so frequency and importance of this phenomenon could be underestimated. According to scarce published data, the oedema could be found in 9% of asymptomatic patients^3^, but our data showed much higher rate of 37.8%. Considering recently found link between occurrence of perianeurysmal oedema after endovascular embolization and recurrence of anurysm^14^, it becomes very important to identify risk factors for the oedema and to modify them with an aim to prevent its occurrence.

Mechanism of perianeurysmal oedema development remains unclear for the time being, but it seems that inflammation plays very important role. Several mechanisms have been proposed to explain perianeurysmal oedema. It may represent a normal healing response after coiling and is probably related by the inflammatory changes. This inflammatory response can be exaggerated in some cases, leading to peryaneurysmal oedema. In other cases, oedema is observed several months after aneurysms treatment and is generally associated with massive recurrence. Oedema can develop also immediately after treatment due to thrombus formation and secondary expansion of aneurysmal sac. It was described around both coiled and uncoiled aneurysms, but the uncoiled ones were invariably thrombosed. If the aneurysm was not thrombosed completely, continuous water-hammering effect against the residual lumen of the aneurysm (aneurysm pulsing) could cause haemorrhage in the aneurysmal wall, triggering inflammation.^15^ This happens mostly in larger aneurysms with wide neck, which explains why in our study the aneurysms with larger volume were more frequently followed with perifocal oedema after embolization. Moreover, larger aneurysms have bigger endothelial surface, which when damaged by coils and blood jet produces larger quantity of autacoids that can initiate process of inflammation.^1^ As a confirmation of these hypotheses, both release of cytokines to cerebrospinal liquor^13^ and inflammation of meninges^12^ were found in patients with perianeurysmal oedema after endovascular coiling. However, although clinical improvement of perianeurysmal oedema was demonstrated in some studies^12^,^13^, others did not confirm such beneficial effect.^15^ In our study pre-embolization administration of corticosteroids did not affect the occurrence of perianeurysmal oedema.

Older patients have higher risk for perianeurysmal oedema after embolization^6^,^8^,^9^,^10^; the patients with oedema in our study were on average 10 years older than the patients without oedema. Hypertension, diabetes mellitus and smoking are also risk factors for perianeurysmal oedema, according to our and some other studies.^6^ This is not surprising, since all these factors adversely affect microcirculation^16^,^17^ leading to increased permeability of capillaries and propensity for inflammation and oedema formation.

Perianeurysmal oedema could be observed after endovascular embolization with any type of coils.^1^–^11^ However, certain studies hypothesized that bioactive coils increase the risk for the oedema up to nine-fold.^8^,^10^,^12^ In our study bare platinum coils did have the smallest rate of perianeurysmal oedema, but the difference was not significant in comparison to the hydrophilic coils. This issue requires further research before any kind of recommendation about choice of coil type could be made.

According to published data the incidence of perianeurysmal oedema is much lower, from a few percentages to 14, 3%.^18^,^19^ However all published cases were symptomatic, presented commonly with headache, malaise or cranial neuropathies. Perianeurysmal oedema is actually much more frequent, but mostly remains asymptomatic (as shown in our study), and is rarely followed by serious complications.

The results of our study suggest that older patients with larger unruptured intracranial aneurysms, who suffer from diabetes mellitus and hypertension, and have the smoking habit, are under much higher risk of having perianeurysmal oedema after endovascular coiling. Such patients should be more strictly controlled by MRI imaging after the embolization, in order to reveal the oedema earlier, and adjust the care for the patient accordingly.

## Figures and Tables

**TABLE 1.** The patients’ characteristics

	**Without perianeurysmal oedema; n = 74**	**With perianeurysmal oedema; n = 45**	**p**
**Age**	46.30 ± 8.26^*^	56.87 ± 9.33^*^	^***^0.0005
**Gender (F)**	26 (35.1)	20 (44.4)	0.414
**Hypertension**	43 (58.1)	44 (97.8)	^***^0.0005
**Diabetes**	8 (10.8)	20 (44.4)	^***^0.0005
**Smoking**	39 (52.7)	34 (75.6)	0.022
**Hypercholesterolemia**	19 (25.7)	30 (66.7)	^***^0.0005
**Corticosteroids before the embolization**	29 (39.2)	13 (28.9)	0.346
**Volume of an aneurysm**	75 (33 – 154)^**^	518 (215 – 898)^**^	^***^0.0005
**The coil type:**			^***^0.006
**- hydrophilic**	22 (52.4%)	20 (47.6%)	
**- platinum**	40 (78.4%)	11 (21.6%)	
**- mixed**	12 (46.2%)	14 (53.8%)	

* = mean ± SD,

** = median (25-th percentile – 75-percentile);

*** = significant difference categorical variables shown as n (%);

**TABLE 2.** Binary logistic regression for occurrence of perianeurysmal oedema as dependent variable

	**Univariate analysis**		**Multivariate analysis**	
	
	p	Odds ratio (CI)	p	Odds ratio (CI)
**Age**	0.0005	1.146 (1.085 – 1.211)		
**Hypertension**	0.001	31.721 (4.144 – 242.784)	0.022	30.599 (1.624 – 576.504)
**Smoking**	0.015	2.774 (1.223 – 6.291)	0.026	5.391 (1.226 – 23.710)
**Diabetes**	0.0005	6.600 (2.577 – 16.901)	0.039	5.336 (1.091 – 26.099)
**Hyperholesterolemia**	0.0005	5.789 (2.575 – 13.015)		
**Volume of the aneurysm**	0.0005	1.010 (1.006 – 1.015)	0.0005	1.013 (1.006 – 1.019)
